# Direct peroral cholangioscopic diagnosis and metal stent failure in a case of refractory arterial hemobilia

**DOI:** 10.1055/a-2686-3414

**Published:** 2025-09-22

**Authors:** Ke Han, Qian Zhao, Yong Sun, Feng Gao, Zhixin Liu, Jiao Liu, Zhuo Yang

**Affiliations:** 1 74643 Department of Endoscopy, General Hospital of Northern Theater Command, Shenyang, China

A 49-year-old man, recently treated for choledocholithiasis and cholangitis with successful stone extraction and nasobiliary drainage, presented with recurrent upper abdominal pain. He had a remote history of iron-deficiency anemia 30 years prior, reportedly cured. On admission, computed tomography (CT) showed inhomogeneous density at the distal common bile duct (CBD) and tortuous vascular shadows in the pancreatic head region ( Fig. 1 ). Laboratory tests revealed severe anemia, elevated liver enzymes, and increased inflammatory markers. Recurrent choledocholithiasis and anemia relapse were initially suspected.

**Fig. 1:**
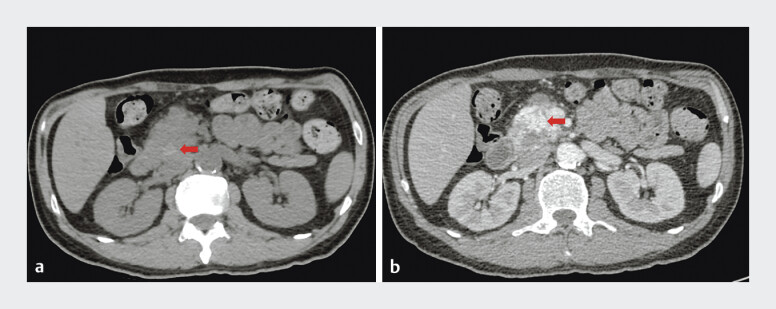
CT showed **a** inhomogeneous density at the CBD and **b** tortuous vascular shadows in the pancreatic head region on admission.

The next day, he developed severe abdominal pain, hematemesis, and hypotension. Emergency upper endoscopy revealed large amounts of fresh blood in the stomach, and duodenoscopy confirmed oozing hemorrhage from the major papilla. ERCP demonstrated a dilated CBD filled with blood clots. Notably, direct peroral cholangioscopy enabled, for the first time, definitive visualization and diagnosis of a pulsatile arterial stump at the distal CBD as the bleeding source, indicating a gastroduodenal artery malformation ( Video 1 ). A fully covered self-expandable metal stent (FCSEMS, 10 mm × 40 mm) was placed for tamponade ( Fig. 2 ). However, rebleeding occurred 6 days later due to stent migration, necessitating replacement with a longer FCSEMS (10 mm × 80 mm), along with nasobiliary drainage and endoscopic clip fixation ( Fig. 3 ).

Cholangioscopic view of a pulsatile arterial stump at the distal CBD.Video 1

**Fig. 2:**
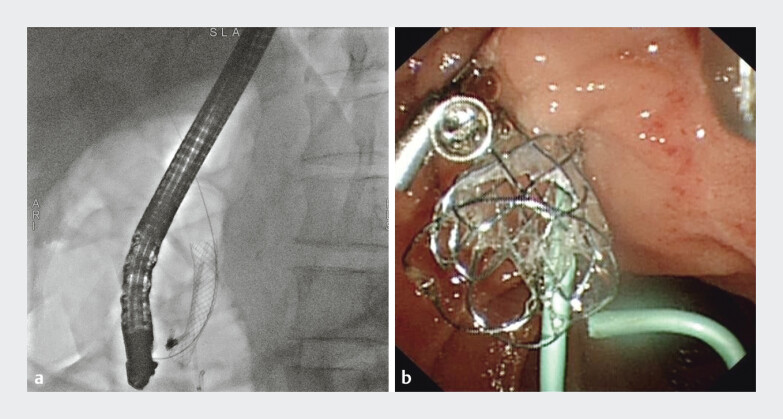
Initial FCSEMS placement (10 mm × 40 mm) for tamponade.

**Fig. 3:**
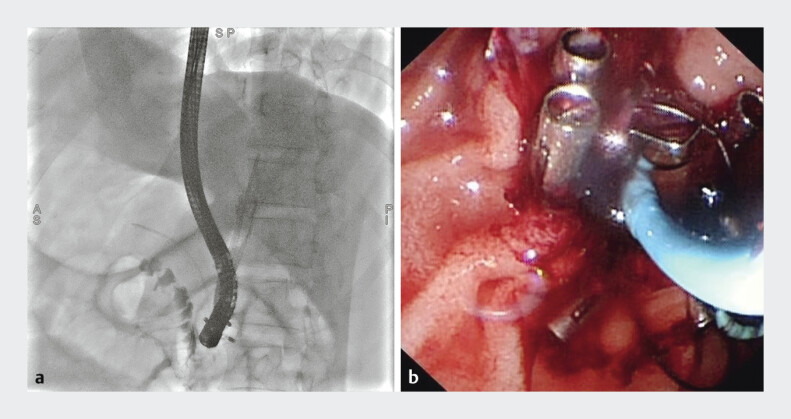
Repeat ERCP with longer FCSEMS (10 mm × 80 mm), nasobiliary drainage, and clip fixation.

Selective angiography revealed a patchy vascular malformation in the gastroduodenal artery territory ( Fig. 4 ), without obvious contrast extravasation. Embolization was performed on two branches of the gastroduodenal artery supplying the malformation. Nevertheless, a third bleeding episode occurred 7 days later. Repeat angiography demonstrated diffuse abnormal vessels in the splanchnic region, precluding further embolization. The patient ultimately underwent pancreaticoduodenectomy.

**Fig. 4:**
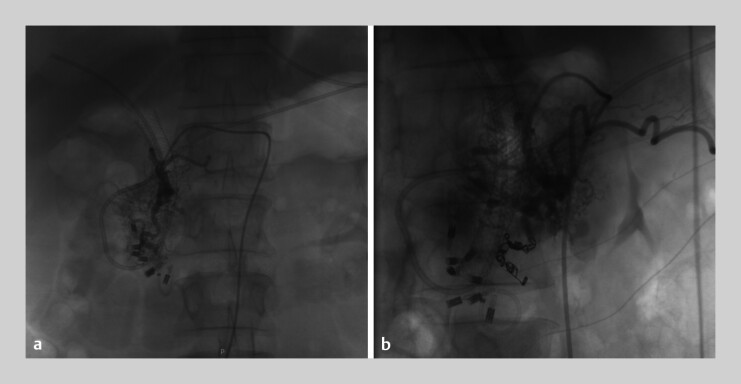
Angiography showed **a** a patchy vascular malformation in the gastroduodenal artery area and **b** two supplying branches were embolized.

To our knowledge, this is the first reported case of arterial hemobilia definitively diagnosed by direct peroral cholangioscopy. While FCSEMS can be effective for hemobilia 1 2 , it may be inadequate for refractory arterial bleeding. Early surgical intervention should be considered when endoscopic and radiologic therapies fail 3 .

Endoscopy_UCTN_Code_CCL_1AF_2AF_3AZ

## Notes

Endoscopy E-Videos https://eref.thieme.de/e-videos
*E-Videos* is an open access online section of the journal *Endoscopy* , reporting on interesting cases and new techniques in gastroenterological endoscopy. All papers include a high-quality video and are published with a Creative Commons CC-BY license. Endoscopy E-Videos qualify for HINARI discounts and waivers and eligibility is automatically checked during the submission process. We grant 100% waivers to articles whose corresponding authors are based in Group A countries and 50% waivers to those who are based in Group B countries as classified by Research4Life (see: https://www.research4life.org/access/eligibility/ ). This section has its own submission website at https://mc.manuscriptcentral.com/e-videos .
